# Comorbidity Differences by Trajectory Groups as a Reference for Identifying Patients at Risk for Late Mortality in Childhood Cancer Survivors: Longitudinal National Cohort Study

**DOI:** 10.2196/41203

**Published:** 2023-03-24

**Authors:** Hyery Kim, Hae Reong Kim, Sung Han Kang, Kyung-Nam Koh, Ho Joon Im, Yu Rang Park

**Affiliations:** 1 Division of Pediatric Hematology and Oncology, Department of Pediatrics Asan Medical Center Children’s Hospital University of Ulsan College of Medicine Seoul Republic of Korea; 2 Department of Biomedical Systems Informatics Yonsei University College of Medicine Seoul Republic of Korea

**Keywords:** cancer survivor, childhood cancer, mortality, morbidity, survival, cancer, children, pediatrics

## Abstract

**Background:**

Childhood cancer has a high long-term morbidity and mortality rate. Five years after the initial cancer diagnosis, approximately two-thirds of childhood cancer survivors experience at least one late complication, with one-quarter experiencing severe, life-threatening complications. Chronic health conditions can impact survivors’ life planning and daily activities, reducing their health-related quality of life. Comprehensive and longitudinal data are required for investigations of national claims data.

**Objective:**

This study aimed to address clinical and health policy interventions and improved survival rates. A comprehensive categorization of the long-term morbidities associated with childhood cancer survivorship is required. We analyzed the trajectory groups associated with long-term mortality among childhood cancer survivors.

**Methods:**

We collected data from a nationwide claims database of the entire Korean population. Between 2003 and 2007, patients diagnosed with and treated for cancer before the age of 20 years were included. With 8119 patients who survived >10 years, 3 trajectory groups were classified according to yearly changes in the number of diagnoses (the lowest in group 1 and the highest in group 3).

**Results:**

The patterns of most comorbidities and survival rates differed significantly between the trajectory groups. Group 3 had a higher rate of mental and behavioral disorders, neoplasms, and blood organ diseases than the other two groups. Furthermore, there was a difference in the number of diagnoses by trajectory groups over the entire decade, and the disparity increased as the survival period increased. If a patient received more than four diagnoses, especially after the fourth year, the patient was likely to be assigned to group 3, which had the worst prognosis. Group 1 had the highest overall survival rate, and group 3 had the lowest (*P*<.001). Group 3 had the highest hazard ratio of 4.37 (95% CI 2.57-7.42; *P*<.001) in a multivariate analysis of late mortality.

**Conclusions:**

Our findings show that the pattern of comorbidities differed significantly among trajectory groups for late death, which could help physicians identify childhood cancer survivors at risk for late mortality. Patients with neoplasms, blood organ diseases, or mental and behavioral disorders should be identified as having an increased risk of late mortality. Furthermore, vigilance and prompt action are essential to mitigate the potential consequences of a child cancer survivor receiving four or more diagnoses within a year.

## Introduction

Childhood cancer survival rates have increased significantly over the last 4 decades [1]. Most patients survive for a long time, with over 500,000 in the United States and 25,000 in Korea [2,3]. However, prolonged survival after treatment is associated with significant long-term morbidity and premature mortality [4]. Approximately two-thirds of childhood cancer survivors develop at least one late complication that occurs or persists 5 years after the initial cancer diagnosis, with one-quarter developing severe, life-threatening late complications [5,6] that have significantly improved over the past 4 decades [1]. The Childhood Cancer Survivor Study (CCSS) found that 54% of long-term childhood cancer survivors developed at least one severe, disabling, life-threatening, or fatal chronic health condition by the age of 50 years, which is five times higher than that of their healthy peers [7]. Chronic health conditions, such as heart failure, secondary neoplasms, and pulmonary dysfunction can be life-threatening, and other health conditions, such as infertility and hearing impairment, can affect survivors’ life planning and daily activities, decreasing their health-related quality of life (HRQoL) [8].

Therefore, monitoring complications and assessing long-term health risks in pediatric and adolescent cancer survivors is essential. In long-term survivors, early intervention may reduce the risk of morbidity due to coexisting conditions. Numerous research groups on a global scale monitor the chronic health condition of childhood cancer survivors, manage their HRQoL [8-11], and develop follow-up strategies using web-based questionnaires [12].

Although previous cohort studies of pediatric cancer survivors elaborated on long-term comorbidities and chronic health conditions, data collection was limited [9,13,14]. First, these studies mainly relied on self-reported questionnaire data and, therefore, identified false-negative outcome events, especially in minor or rare complications that survivors do not report. Second, unlike Korea’s single-payer health care system, health care providers in other countries are subject to changes in individual patients, making the collection of complete medical records difficult [15]. Third, existing cohorts have limitations covering a long period or accurately representing the general population. Lastly, previous studies primarily described the first occurrence of complications and did not show morbidity trends over time.

The Korean National Health Insurance Service (NHIS) is South Korea’s universal health insurance coverage system [16]. It includes detailed treatment practices and prescriptions based on the fee-for-service payment model and the health information of all South Korean citizens enrolled in national medical insurance [16]. Patients with cancer can receive special government support for medical expenses in South Korea; therefore, once diagnosed, patients with cancer must be registered with the NHIS with a special code and cancer diagnosis so that we can identify their claims data [17]. The NHIS has constructed and operated large-capacity health care big data systems, providing researchers with a wide range of secure health care data for research purposes. The NHIS collects longitudinal patient data that can be used for longitudinal temporal studies, such as the analysis of long-term cancer survivors. Consequently, active longitudinal and epidemiological studies of patients with cancer are being conducted using NHIS data [18-20].

We analyzed long-term health-related conditions using the nationwide claims database of Korea, which contains medical records for approximately 97% of the South Korean population. This study aimed to investigate childhood cancer survivors’ temporal diagnostic patterns and assess late mortality using a trajectory group-based method.

## Methods

### Data Source

We obtained data from the NHIS, a government insurer in Korea that provides universal health insurance to approximately 97% of Koreans [17]. The NHIS is a universal health insurance system that includes detailed treatment practices and prescriptions based on the fee-for-service payment model and the health information of all Korean citizens who have signed up for national medical insurance [16]. Data included public data on health care use, such as disease diagnoses, drug prescriptions, and procedures. National health examination results for the entire Korean population, including smoking habits, alcohol consumption, physical measurements, body measurements, and demographics as well as socioeconomic variables such as age, sex, income rank, household location, disability, and mortality. Diagnoses were coded in accordance with the *International Statistical Classification of Disease, Tenth Revision* (*ICD-10*) within health insurance claims data [16,21]. The NHIS data were linked to death statistics from Statistics Korea based on the resident numbers of patients [22]. We collected death information from all childhood cancer survivors for this study. Public users can access all data from NHIS and Korean Statistics Information Services for research purposes.

### Study Population

We identified 71,323 patients from the NHIS database who were younger than 20 years and had any cancer codes (based on the *ICD-10*) between 2002 and 2017. Clinically representative diagnostic groups were defined for analysis (Multimedia Appendix 1). There were 549 claim codes for treatment (ie, chemotherapy, radiotherapy, hematopoietic stem cell transplantation [HCT]; Multimedia Appendix 2). Surgery was not considered treatment because of this ambiguity. The time of earliest cancer diagnosis was defined as the onset of the earliest diagnosis in patients who were prescribed any treatment within 1 month of the initial diagnosis.

**Figure 1:**
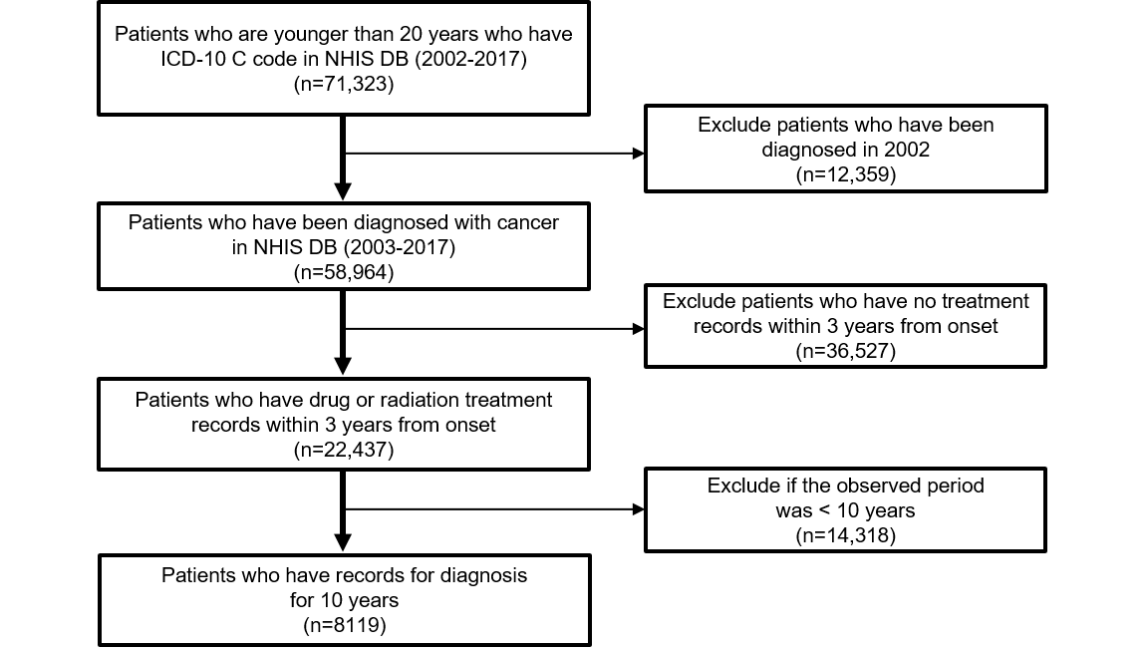
Patient selection flowchart. DB: database; ICD-10: International Classification of Diseases, 10th Revision; NHIS: National Health Insurance Service.

A total of 12,359 patients diagnosed in 2002 and 36,527 patients without treatment records within 3 years from the onset date were excluded (Figure 1). Subsequently, 8119 patients who had survived for >10 years were selected for the final analysis.

### Analyzing Trajectory Groups

For trajectory analysis, we used each patient’s total number of diagnoses per year as an input variable. We used a square root transformation to calculate the total number of diagnoses. Age, sex, year of onset and diagnosis, duration of treatment exposure, total number and type of treatment, type of care, and socioeconomic status were used to identify differences between the trajectory groups. The most significant diagnosis was entered into the “Main Diagnosis” field, while other diagnoses were entered into the “Subdiagnosis” field. Only those entered in the “Main Diagnosis” section were used for analysis. We pooled all main diagnostic codes from each patient’s hospital visits during the survival period and analyzed the diagnoses by excluding primary cancer as a comorbidity.

We used group-based latent trajectory modeling (GBTM) on longitudinal data of childhood cancer survivors to identify homogenous subgroups of a population with similar patterns of outcome change over time [23]. Response values were estimated using finite mixture models in GBTM. The GBTM was fitted with a censored normal distribution, assuming that the total number of diagnoses per year in the claim data follows a normal distribution with a limited scale [24]. The following criteria were used for selecting the optimal number of groups: the lowest value in the Bayesian information criteria, the average posterior probability of group assignment (≥0.7), and group size such that ≥5% of the study sample was assigned to one trajectory group [25]. The trajectory group was assessed using the Proc Traj procedure in SAS 9.4 [26] (SAS Institute).

### Statistical Analysis

Trajectory group characteristics are presented as means with SDs for normally distributed characteristics or as medians with IQRs for skewed distribution. The chi-square tests for categorical variables and one-way ANOVA for continuous variables were used to compare the characteristics of the trajectory groups. Kaplan-Meier methods and log-rank tests were used for survival analysis. Cox proportional hazard models with 95% CIs were estimated to investigate differences [27]. The absolute standardized difference (ASD) is a standard measure for comparing values before and after propensity matching. However, pairwise comparisons were performed for the three study groups to identify intergroup differences based on the maximum value. According to the guidelines, the two groups are different from each other by 0.1 [28]. We used the Stddiff package in R (version 3.5.3; R Foundation for Statistical Computing) to calculate ASD. *P* values <.05 on both sides were considered statistically significant.

### Ethics Approval

This study was conducted in accordance with the principles of the Declaration of Helsinki and approved by the institutional review board of the Asan Medical Center in Seoul, Republic of Korea (IRB 2019-0915). All KNHIS patient records were anonymized to ensure patient confidentiality. Because the analysis used anonymous data, the institutional review board waived the requirement for informed consent.

## Results

### Trajectory Groups for Pediatric Patients With Cancer

In the 10-year survivor cohort (n=8119; Multimedia Appendices 3 and 4), 3 trajectory groups were selected with the best fit. The first group (n=3104, 38.2%) had relatively smaller changes in the number of diagnoses over time and was thus referred to as the “relatively stable group (group 1)” (red line; Multimedia Appendix 4). The second group (n=4148, 51.1%) was referred to as the “moderately decreasing group (group 2)” (green line), and the remaining population (n=867, 10.7%) was referred to as the “rapidly decreasing group (group 3)” (blue line), which had the most diagnoses and a rapidly decreasing trend over time.

### Characteristics by Trajectory Groups

The median age at onset significantly decreased in groups 1 to 3 (*P*<.001; Table 1). Lymphoid leukemia was found in 158 of 867 (18.2%) patients of group 3 patients and 838 of 3104 (20.2%) patients in group 2; however, only 264 of 3104 (8.5%) patients of group 1 (*P*<.001). The top five onset diagnoses were “secondary and unspecified malignant neoplasm of lymph nodes.” The median duration of treatment exposure and the total number of prescriptions increased significantly in groups 1 to 3 (*P*<.001).

The use of radiotherapy and the proportion of patients with any HCT increased significantly from group 1 to 3 (radiotherapy: 52.2%, 70.4%, 76.2%; *P*<.001, HCT: 4.1%, 11.2%, 21.6%; *P*<.001, respectively).

In terms of the onset year, 46.1% of group 1 and 21.5% of group 3 were diagnosed between 2003 and 2004 (*P*<.001). In terms of the temporal patterns of each categorical variable, chemotherapy use decreased, while chemotherapy with radiotherapy or HCT steadily increased (Multimedia Appendix 5).

**Table 1:** Characteristics by trajectory groups for 10 years of follow-up with childhood cancer survivors.

Category			Group 1 (n=3104)		Group 2 (n=4148)		Group 3 (n=867)		Total (n=8119)		*P* value^a^	
Onset age (years), median (IQR)			12 (7.0-16.0)		7 (3.0-13.0)		3 (1.0-10.0)		9 (4.0-14.0)		<.001	
**Sex, n (%)**												.04
	Male	1818 (58.6)		2308 (55.6)		496 (57.2)		4622 (56.9)				
	Female	1286 (41.4)		1840 (44.4)		371 (42.8)		3497 (43.1)				
**Top 5 onset diagnoses in total, n (%)**												
	Lymphoid leukemia	264 (8.5)		838 (20.2)		158 (18.2)		1260 (15.5)		.05		
	CNS^b^ tumor	363 (11.7)		501 (12.1)		161 (18.6)		1025 (12.6)		.29		
	NHL^c^	347 (11.2)		402 (9.7)		76 (8.8)		825 (10.2)		.85		
	Myeloid leukemia	208 (6.7)		339 (8.2)		56 (6.5)		603 (7.4)		.88		
	Secondary and unspecified malignant neoplasm of the lymph nodes	296 (9.5)		239 (5.8)		33 (3.8)		568 (7.0)		.25		
**Claims records, n (%)**												<.001
	Inpatient	17,923 (5.0)		59,236 (6.0)		26,007 (6.1)		103,166 (5.8)				
	Outpatient	342,558 (94.6)		932,153 (93.7)		399,001 (93.4)		1,673,712 (93.8)				
	Emergency	1579 (0.4)		3106 (0.3)		2190 (0.5)		6875 (0.4)				
Social economy status^d^, median (IQR)			14 (9.0-17.0)		14 (9.0-17.0)		13 (9.0-16.0)		14 (9.0-17.0)		<.001	
Treatment exposure^e^ (days), median (IQR)			249 (0-1027)		935 (240-1835)		1506 (464-3266)		651 (120-1655)		<.001	
Total number of prescriptions for treatment, median (IQR)			7 (2-43)		45 (6-114)		74 (15-168)		27 (3-87)		<.001	
**Types of treatment, n (%)**												
	CT^f^ + RT^g^	1268 (40.9)		2278 (54.9)		450 (51.9)		3996 (49.2)		.11		
	CT only	1479 (47.6)		1223 (29.5)		197 (22.7)		2899 (35.7)		<.001		
	RT only	231 (7.4)		178 (4.3)		25 (2.9)		434 (5.3)		.32		
	CT + RT + allo^h^ HCT^i^	69 (2.2)		288 (6.9)		94 (10.8)		451 (5.6)		.05		
	CT + RT + auto^j^ HCT	53 (1.7)		175 (4.2)		92 (10.6)		320 (3.9)		.02		
	CT + allo HCT	2 (0.1)		0 (0.0)		0 (0.0)		2 (0.02)		.91		
	Allo HCT only	2 (0.1)		0 (0.0)		1 (0.1)		3 (0.04)		.95		
**Summary of treatment, n (%)**												
	Any RT	1621 (52.2)		2919 (70.4)		661 (76.2)		5201 (64.1)		<.001		
	Any HCT	126 (4.1)		463 (11.2)		187 (21.6)		776 (9.6)		<.001		
**Year of onset, n (%)**												<.001
	2003-2004	1431 (46.1)		1407 (33.9)		186 (21.5)		3024 (37.2)				
	2005-2006	937 (30.2)		1437 (34.6)		347 (40.0)		2721 (33.5)				
	2007-2008	736 (23.7)		1304 (31.4)		334 (38.5)		2374 (29.2)				

^a^Calculated using the chi-square test (categorical variables) or one-way ANOVA (numerical variables).

^b^CNS: central nervous system.

^c^NHL: non-Hodgkin lymphoma.

^d^Socioeconomic status refers to the 20th quartile by insurance fee, and 1255 patients did not have the quartile information.

^e^Treatment exposure was defined as the duration from the first date to the last date when any chemotherapeutic agents or radiotherapy codes were prescribed.

^f^CT: chemotherapy.

^g^RT: radiotherapy.

^h^Allo: allogeneic.

^i^HCT: hematopoietic stem cell transplantation.

^j^Auto: autologous.

### Comorbidity by Trajectory Groups

**Figure 2:**
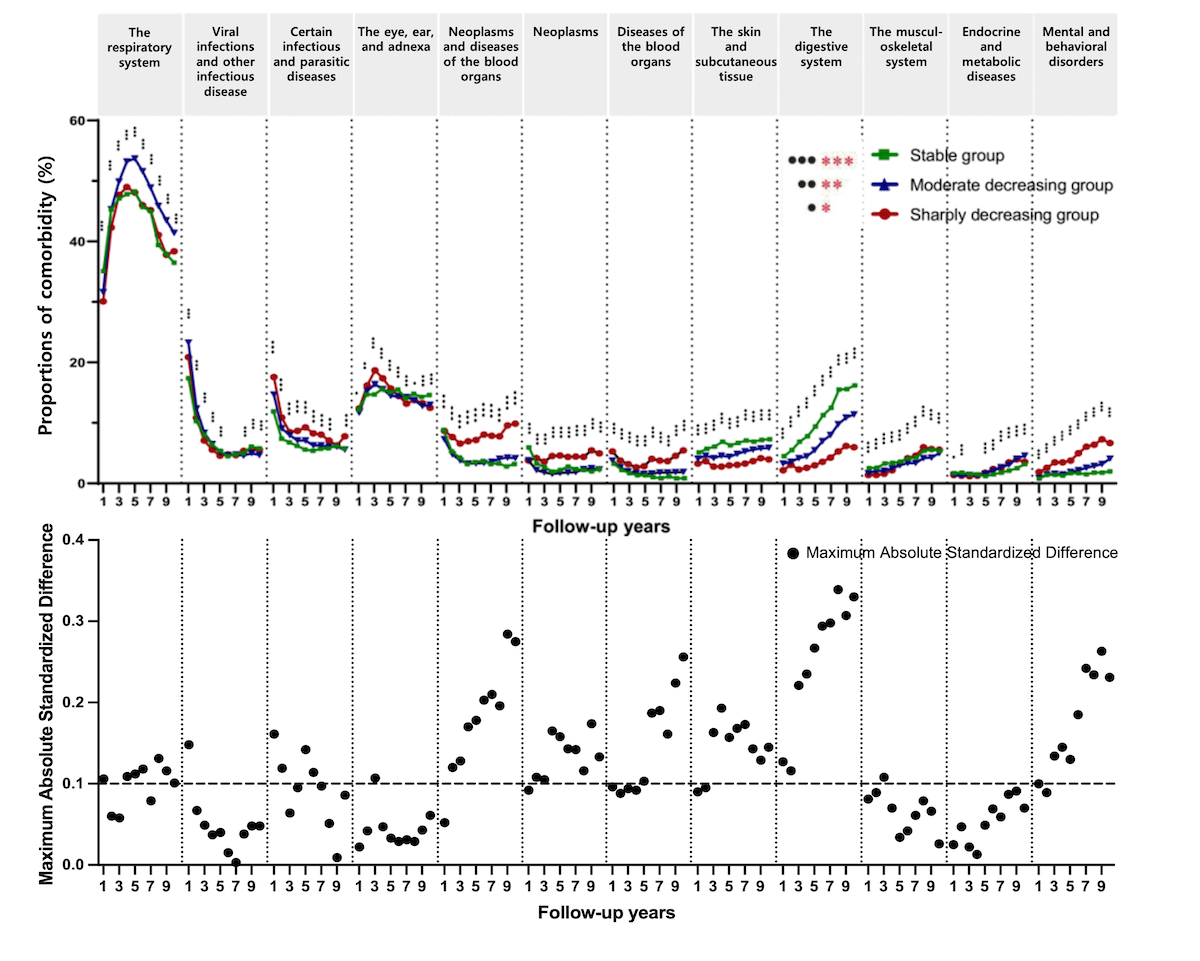
The prevalence of the top 10 comorbidities of pediatric cancer survivors by follow-up year and a plot of the maximum absolute standardized difference between two groups by follow-up year in the top 10 comorbidities, presented pairwise. Pairwise *P* values for two of the three groups are presented as follows: *<.05, **<.01, and ***<.001. No *P* values were corrected.

Figure 2 shows the proportion of each diagnosis in relation to the total yearly numbers of the top 10 comorbidities (Multimedia Appendix 6). The annual incidence showed three major trends: early and decreased midterm surge and continuously decreasing and growing proportions. Infection-related disorders (eg, certain infections, parasitic diseases, viral infections, and other infectious diseases) have declined since the beginning (Figure 2 and Multimedia Appendix 7). Although respiratory disorders were the most common comorbidities at all times, the proportion of diseases in the eye, ear, adnexa, or respiratory system showed a midterm surge at approximately 3-5 years after diagnosis. However, endocrine or metabolic, mental or behavioral, digestive, musculoskeletal, and skin-related disorders have shown increasing trends over the last 10 years. The proportions of comorbidities were statistically different among the three groups at most follow-up periods in the top 10 comorbidities. Specifically, mental and behavioral disorders predominantly increased in group 3 within the 10 years and were statistically significant among trajectory groups for 10 years. Furthermore, in group 3, neoplasms or diseases of the blood organs (D00-D89) showed a unique trend, decreasing in the first 4 years and increasing thereafter. In groups 1 and 2, there was a decreasing pattern, and the differences were statistically significant at all times. When the D code was divided into neoplasm (D00-D49) and disease of the blood organs (D50-D89) for statistical analysis, group 3 was statistically substantially higher during the entire period.

When ASD was used to evaluate the judgment, it was more sensitive than the chi-square test results. By the follow-up year, ASD of mental and behavioral disorders, neoplasms, diseases of the blood organs, skin and subcutaneous tissue, and the digestive system showed increasing trends above the threshold of 0.1, demonstrating *overt* differences in the incidence of comorbidities between each group (Figure 2 and Multimedia Appendix 7). Over 10 years, the prevalence of mental and behavioral disorders, neoplasms, and diseases of the blood organs (also known as neoplasm and blood organ disease) gradually increased in group 3. The prevalence of ASD in these diseases was relatively high in groups 3 and 1. For the past 10 years, skin, subcutaneous tissue, and digestive system diseases increased, particularly in group 1. There was a significant difference between groups 3 and 1.

### Survival Outcome by Trajectory Groups

**Figure 3:**
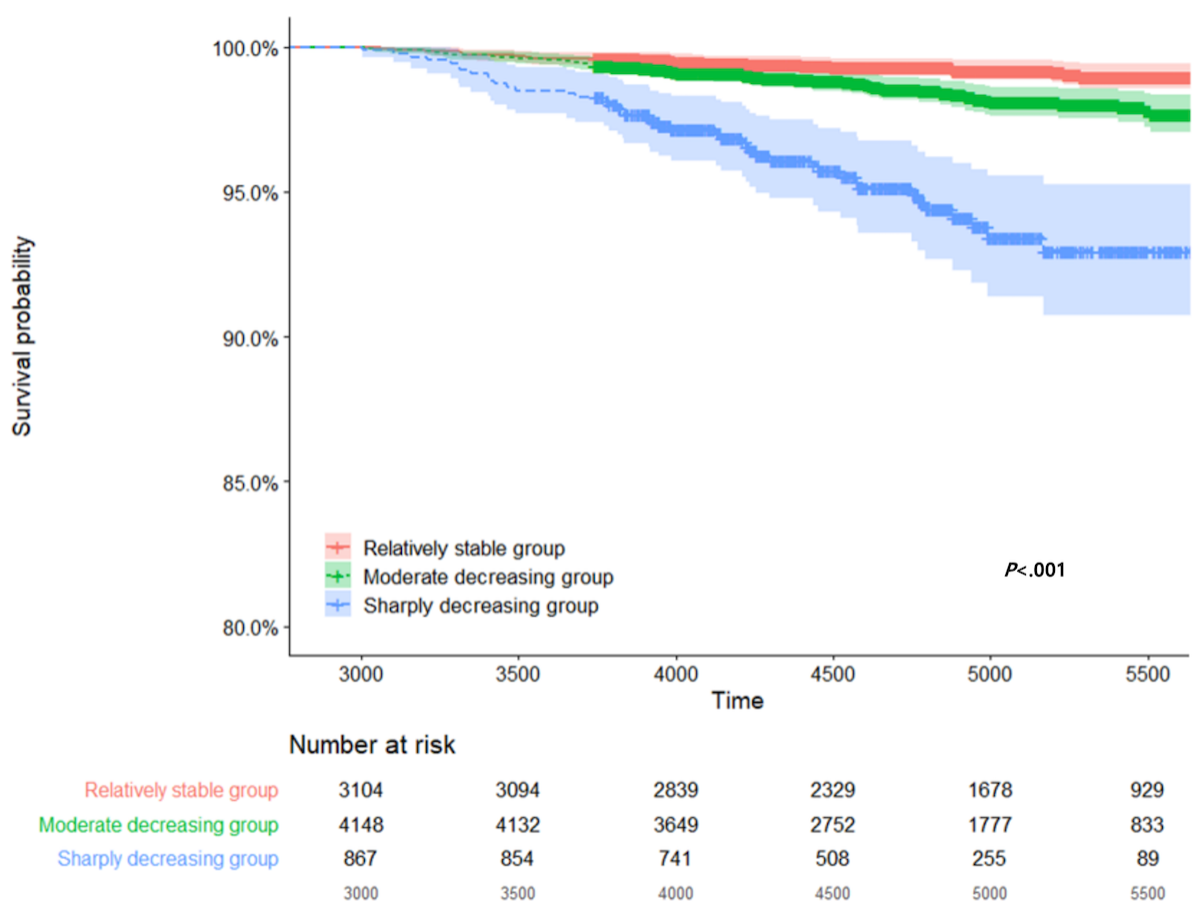
Kaplan-Meier plots for overall survival by trajectory groups for 10-year follow-up. Overall survival was highest in group 1 and lowest in group 3 (*P*<.001).

In terms of overall survival, it was confirmed that the mortality rate increased more rapidly in group 3 than in group 1. In particular, it was observed that the difference in mortality rate increased more rapidly from 3700 days in group 3 than in groups 1 and 2, which was a statistically significant result (*P*<.001; Figure 3). Multivariate analysis was performed after adjusting for sex, age at onset (5 years), and treatment exposure duration.

Group 3 had the highest risk as determined by the trajectory method. The hazard ratio (HR) was 4.37 (95% CI 2.57-7.42; *P*<.001; Table 2). Group 2 had an HR of 1.68 (95% CI 1.05-2.7; *P*=.03) compared with group 1. The age was calculated by dividing it by 5 years. As a result, it was confirmed that the patient’s age when compared to 5 years of age increased the risk of overall survival. The HR for overall survival was 1.53 (95% CI 0.91-2.59; *P*=.11) when compared to the group younger than 5 years (reference group). However, it was not statistically significant. The HRs or overall survival were 2.68 (95% CI 1.64-4.39; *P*<.001) and 2.57 (95% CI 1.57-4.22; *P*<.001), respectively, and had a significant effect on survival when compared to the reference group. The result of the multivariate survival analysis was adjusted for sex and treatment duration. Central nervous system tumor was the most common cause of death at the time of death (Multimedia Appendix 8).

**Table 2:** HRs for all-cause death by trajectory groups.

Cohort and variables			Univariate					Multivariate^a^		
			HR^b^ (95% CI)		*P* value		HR (95% CI)			*P* value
**10-year**										
	Group 1	1 (reference)		N/A^c^		1 (reference)			N/A	
	Group 2	2.11 (1.33-3.36)		.001		1.68 (1.05-2.7)			.03	
	Group 3	7.32 (4.47-12.01)		<.001		4.37 (2.57-7.42)			<.001	
	Onset age (<5 years old)	1 (reference)		N/A		1 (reference)			N/A	
	Onset age (≥5 years and <10 years)	1.2 (0.72-2.02)		.48		1.53 (0.91-2.59)			.11	
	Onset age (≥10 years and <15 years)	1.66 (1.03-2.69)		.04		2.68 (1.64-4.39)			<.001	
	Onset age (≥15 years)	1.67 (1.04-2.69)		.04		2.57 (1.57-4.22)			<.001	
	Sex (reference: male)	0.57 (0.39-0.82)		.003		N/A			N/A	
	Treatment exposure duration	1.001 (1.001-1.001)		<.001		N/A			N/A	

^a^Adjusted for sex and duration of treatment.

^b^HR: hazard ratio.

^c^N/A: not applicable.

## Discussion

This study evaluated the 10-year trajectories of childhood cancer survivors nationwide. Using group-based trajectory analysis, we analyzed all hospital visits due to any health impairments. We demonstrated that the risk groups for long-term mortality could be well classified using the entire spectrum of diagnoses as a surrogate for the cumulative burden of late complications. Our study found that the pattern of cumulative comorbidities during the survival periods significantly differed according to the trajectory group; physicians could use this to identify patients at risk for late mortality in childhood cancer survivors.

The NHIS database was used in this study, which can complement the limitations of previous studies on cancer survivors. The NHIS database is one of the world’s largest claims data sets. It centralizes all claims data from the Korean population regardless of age or region, distinguishing it from the Medicare and Medicaid programs in the United States. This reduces the possibility of selection bias [29]. This universal health care provider system facilitates the collection of populationwide longitudinal data, which makes it helpful in analyzing the long-term outcomes of childhood cancer survivors.

The St Jude Lifetime Cohort Study (SJLIFE) recently reported the cumulative disease burden of 168 chronic health conditions grouped into 48 condition-specific categories for childhood cancer survivors [9]. SJLIFE reported that the survivors advancing age changed the landscape of the dominant comorbid conditions. In this report, the cumulative burden of chronic health conditions per individual was differentiated by the initial cancer types of the survivors. This report from SJLIFE could provide general health practitioners and clinical investigators with references for the expected cumulative burden of comorbidities in specific survivor groups.

Although late mortality is as important as a chronic disease in childhood cancer survivors, there are no direct references to late mortality. Additionally, psychiatric diseases were not analyzed.

Our findings suggest that the pattern of comorbidities can predict high-risk groups for late mortality. Childhood cancer survivors are at risk for adverse psychological outcomes according to the CCSS study of 6199 patients, depression had a prevalence of 11.4% after a median follow-up duration of 7.8 years [30]. Survivors have an 80% higher risk of developing clinically significant mental health symptoms than their siblings [31].

According to the results of this study, high-risk groups with low survival rates are likely to include childhood cancer survivors who receive more than a certain number of diagnoses annually during long-term follow-up. Our study showed that mental and behavioral disorders could increase the risk of late mortality due to other severe late complications. Therefore, survivors who often visit hospitals for mental or behavioral disorders should be closely monitored for late mortality. In addition, neoplasms or blood organ diseases were common in group 3, and these conditions were associated with abnormal hematologic findings unrelated to the malignancy. Therefore, such blood organ diseases might result from other severe health conditions or complications of ongoing treatment.

Furthermore, there was a difference in the number of diagnoses by trajectory group not only in the first year but throughout the decade (Multimedia Appendix 4). As the duration of survival increased, so did the disparity. For the first 1-3 years, there was no significant difference in the number of average diagnoses between groups; however, if the patient received more than four diagnoses, especially after the fourth year, the patient was strongly suspected to be in group 3, which had the worst prognosis. Consequently, if a childhood cancer survivor receives four or more diagnoses in a year, close monitoring and early intervention for complications are necessary.

Factors associated with the high-risk trajectory group in this study were younger age at primary cancer diagnosis, longer treatment duration, and a recent treatment period with radiotherapy or HCT. The duration of treatment and the number of treatment codes were proportional to the overall loading of the treatment; therefore, a higher loading of the treatments was invariably related to a higher risk of mortality.

Reports on the age at primary cancer diagnosis and its relationship to subsequent cancers are contradictory [32-34]. Generally, younger age at the time of radiotherapy or HCT is associated with an increased risk of complications and subsequent cancers [35,36]. Particularly, testicular cancer and Hodgkin disease are risk factors for developing cardiovascular disease, secondary malignancies, and death from noncancerous causes at a younger age [37].

Treatment timing distinguished the trajectory groups in this study. Other studies have found that previously treated patients had a higher risk of long-term complications and mortality than those receiving treatment [14,38,39]. Contrary to previous studies, our study found that more recently treated survivors had poorer survival outcomes than those previously treated, possibly because our study included data from 2003 rather than the 1990s. Moreover, since 2003, radiotherapy and HCT have been increasingly used with other treatment modalities. The increasing use of alternative donors [40] and the introduction of tandem autologous HCT in patients with high-risk neuroblastoma and medulloblastoma may have contributed to the recent increase in HCT [41,42]. Therefore, more recent survivors are likely to have received intensive treatment.

Although this study showed a novel approach to examining nationwide longitudinal data using trajectory analysis, it had several limitations. First, because this data set was established for claims and reimbursements, there may be differences between the actual disease and the claimed diagnosis. Second, this study did not include a healthy control group. Third, it could be argued that the difference in survival between the trajectory groups is due to the high frequency of recurrence or secondary cancer in the high-risk group rather than comorbidities. Finally, claims databases are incapable of distinguishing between cancer remission and recurrence because the recurrence diagnosis and treatment discontinuation needs to be clearly defined. This may have led to skewed results in identifying trajectory groups with increased morbidity and mortality due to protracted chemotherapy-related comorbidities.

In conclusion, our study is the first to show that the risk groups for long-term mortality in childhood cancer survivors can be grouped using the entire spectrum of diagnosed diseases as a surrogate for the cumulative burden of late complications. We demonstrated that clinically severe health conditions and hospital visits due to health impairments could significantly impact long-term survival. Even without a primary cancer diagnosis, medical practitioners should pay attention to the subpopulation of patients with increased or specific comorbidities.

## Appendix

Multimedia Appendix 1 Representative diagnostic groups for the International Classification of Diseases codes.

Multimedia Appendix 2 List of claiming codes in the Korean National Health Insurance Program for cancer treatments.

Multimedia Appendix 3 Tabulated Bayesian information criteria (BIC) and 2∆BIC.

Multimedia Appendix 4 Group-based trajectory graphs based on the transformed numbers of diagnosis by follow-up year.

Multimedia Appendix 5 Comparison of onset diagnosis and the types of treatment every year after diagnosis.

Multimedia Appendix 6 International Classification of Diseases codes for the top 10 comorbidities.

Multimedia Appendix 7 Absolute standardized differences between comorbidities of the three groups by follow-up year (for 10 years).

Multimedia Appendix 8 Diagnosis at the time of death.

## Abbreviations

ASD: absolute standardized difference
CCSS: Childhood Cancer Survivor Study
GBTM: group-based latent trajectory modeling
HCT: hematopoietic stem cell transplantation
HR: hazard ratio
HRQoL: health-related quality of life
ICD-10: International Statistical Classification of Disease, Tenth Revision
NHIS: National Health Insurance Service
SJLIFE: St Jude Lifetime Cohort Study

## Data Availability

The data are not publicly available due to privacy or ethical restrictions.
