# Supplementary material for: Comorbidity Differences by Trajectory Groups as a Reference for Identifying Patients at Risk for Late Mortality in Childhood Cancer Survivors: Longitudinal National Cohort Study
Source: JMIR Public Health Surveill. 2023 Mar 24;9:e41203. doi: 10.2196/41203 (PMC10131914; doi:10.2196/41203)
Supplement: Multimedia Appendix 1 [file publichealth_v9i1e41203_app1.docx]

**Multimedia Appendix 1.** Representative diagnostic groups for the International Classification of Diseases (ICD) codes

| **ICD code** | **Detailed diagnosis** | **Representative diagnostic group** |
| --- | --- | --- |
| C91 | Lymphoid leukemia | Lymphoid leukemia |
| C92 | Myeloid leukemia | Myeloid leukemia |
| C93 | Monocytic leukemia | Monocytic leukemia |
| C81 | Hodgkin lymphoma | Hodgkin lymphoma |
| C82 | Follicular lymphoma | Non-Hodgkin lymphoma |
| C83 | Non-follicular lymphoma |  |
| C84 | Mature T/NK-cell lymphoma |  |
| C85 | Other and unspecified types of non-Hodgkin lymphoma |  |
| C86 | Other specified types of T/NK-cell lymphoma |  |
| C70 | Malignant neoplasm of the meninges | Central nervous system tumor |
| C71 | Malignant neoplasm of the brain |  |
| C72 | Malignant neoplasm of the spinal cord, cranial nerves, and other parts |  |
| C74 | Malignant neoplasm of the adrenal gland | Neuroblastoma |
| C69 | Malignant neoplasm of the eye and adnexa | Retinoblastoma |
| C64 | Malignant neoplasm of the kidney, except the renal pelvis | Renal tumor |
| C40 | Malignant neoplasm of the bone and articular cartilage of the limbs | Bone tumor |
| C41 | Malignant neoplasm of the bone and articular cartilage of other and unspecified sites |  |
| C22.2 | Hepatoblastoma | Hepatoblastoma |
| C49 | Malignant neoplasm of other connective and soft tissues | Rhabdomyosarcoma |

| Unless specified otherwise, subclassification diagnoses were included into the upper classification (e.g., C83.1🡪 C83).  NK, natural killer. |
| --- |
