# Supplementary material for: Comorbidity Differences by Trajectory Groups as a Reference for Identifying Patients at Risk for Late Mortality in Childhood Cancer Survivors: Longitudinal National Cohort Study
Source: JMIR Public Health Surveill. 2023 Mar 24;9:e41203. doi: 10.2196/41203 (PMC10131914; doi:10.2196/41203)
Supplement: Multimedia Appendix 2 [file publichealth_v9i1e41203_app2.docx]

**Multimedia Appendix 2.** List of claiming codes in the Korean National Health Insurance Program for cancer treatments.

| Chemotherapeutic agents | | Code for Korean National Health Insurance Service |
| --- | --- | --- |
|  | Abiraterone | 620402ATB |
|  | Afatinib | 626101ATB–626103ATB |
|  | Aflibercept | 658801BIJ, 658802BIJ |
|  | Aldesleukin | 104501BIJ |
|  | Alectinib | 656201ACH |
|  | Altretamine | 358301ACH |
|  | Amsacrine | 108801BIJ |
|  | Anastrozole | 109001ATB |
|  | Anhydrous | 588201ATB–588207ATB |
|  | Arsenic | 588430BIJ |
|  | Arsenictrioxide | 588401BIJ |
|  | Atezolizumab | 657701BIJ |
|  | Axitinib | 621001ATB, 621002ATB |
|  | Azacitidine | 484301BIJ, 484302BIJ |
|  | Belotecan | 452801BIJ |
|  | Bendamustine | 614601BIJ, 614602BIJ |
|  | Bevacizumab | 554330BIJ, 554331BIJ, 554301BIJ, 554302BIJ |
|  | Bicalutamide | 117201ATB, 117202ATB |
|  | Bleomycin | 118001BIJ |
|  | Blinatumomab | 647701BIJ |
|  | Bortezomib | 463301BIJ, 463303BIJ |
|  | Brentuximab | 624501BIJ |
|  | Brigatinib | 675701ATB |
|  | Buserelin | 120401CSI |
|  | Busulfan | 120601ATB, 120602BIJ, 120630BIJ |
|  | Cabazitaxel | 613901BIJ |
|  | Cabozantinib | 666401ATB–666403ATB |
|  | Capecitabine | 122701ATB, 122702ATB |
|  | Carboplatin | 123701BIJ–123704BIJ, 123706BIJ–123708BIJ, 123730BIJ–123736BIJ |
|  | Carfilzomib | 647801BIJ, 647802BIJ |
|  | Carmofur | 124301ATB |
|  | Ceritinib | 634401ACH |
|  | Cetuximab | 556401BIJ, 556402BIJ, 556430BIJ, 556431BIJ |
|  | Chlorambucil | 130901ATB |
|  | Cisplatin | 134501BIJ–134503BIJ, 134530BIJ–134534BIJ |
|  | Cladribine | 134830BIJ |
|  | Clofarabine | 614301BIJ, 614330BIJ |
|  | Crizotinib | 617501ACH, 617502ACH |
|  | Cyclophosphamide | 139001ATB, 139003BIJ–139005BIJ |
|  | Cyclosporine | 194701ACS, 194702ACS |
|  | Cytarabine | 139601BIJ–139605BIJ, 139609BIJ, 139630BIJ–139638BIJ |
|  | Dabrafenib | 663101ACH, 663102ACH |
|  | Dacarbazine | 139901BIJ–139903BIJ |
|  | Dactinomycin | 140101BIJ |
|  | Daratumumab | 667101BIJ, 667102BIJ |
|  | Dasatinib | 493301ATB–493305ATB |
|  | Daunorubicin | 140601BIJ |
|  | Decitabine | 495601BIJ, 495602BIJ |
|  | Degarelix | 624401BIJ, 624402BIJ |
|  | Diethylstilbestrol | 364701ATB |
|  | Docetaxel | 148301BIJ, 148302BIJ, 148304BIJ, 148306BIJ, 148309BIJ, 148310BIJ, 148341BIJ–148351BIJ |
|  | Doxifluridine | 149301ACH, 149302ACH |
|  | Doxorubicin | 149401BIJ–149406BIJ, 149430BIJ–149435BIJ |
|  | Enocitabine | 151901BIJ |
|  | Enzalutamide | 627401ACS |
|  | Epirubicin | 152701BIJ–152704BIJ, 152730BIJ–152733BIJ |
|  | Eribulin | 621330BIJ |
|  | Erlotinib | 477401ATB–477403ATB |
|  | Estramustine | 155101ACH |
|  | Etoposide | 157101BIJ, 157102ACH, 157103BIJ–157108BIJ, 157130BIJ–157136BIJ |
|  | Everolimus | 485605ATB–485607ATB |
|  | Exemestane | 358401ATB |
|  | Filgrastim | 158901BIJ–158903BIJ, 158930BIJ–158936BIJ |
|  | Fludarabine | 160101BIJ, 160102ATB, 477801ATB |
|  | Fluorouracil | 161401BIJ–161404BIJ, 161430BIJ–161432BIJ |
|  | Fulvestrant | 568931BIJ |
|  | Ganciclovir | 164602BIJ, 164630COO |
|  | Gefitinib | 453001ATB |
|  | Gemcitabine | 164901BIJ–164903BIJ, 164930BIJ–164932BIJ |
|  | Goserelin | 167201BIJ, 167202BIJ |
|  | Heptaplatin | 168801BIJ, 168802BIJ |
|  | Hydroxyurea | 172001ACH, 172002ACH |
|  | Ibrutinib | 628101ACH |
|  | Idarubicin | 173002ACH, 173002BIJ |
|  | Ifosfamide | 173301BIJ |
|  | Imatinib | 412701ATB–412704ATB |
|  | Immunoglobulin | 174301BIJ |
|  | Interferon | 175502BIJ, 175504BIJ, 175530BIJ, 175601BIJ–175608BIJ, 175630BIJ, 175631BIJ |
|  | Irinotecan | 177401BIJ–177408BIJ, 177430BIJ–177437BIJ |
|  | Lapatinib | 507501ATB |
|  | L-Asparaginase | 181401BIJ, 181403BIJ |
|  | Lenalidomide | 588201ACH–588207ACH |
|  | Lenvatinib | 645201ACH, 645202ACH |
|  | Letrozole | 182201ATB |
|  | Leuprolide | 182607BIJ, 182609BIJ |
|  | Leuprorelin | 182601BIJ–182611BIJ, 182630BIJ |
|  | Medroxyprogesterone | 188905ATB |
|  | Megestrol | 189301ASS, 189301ATB, 189302ATB, 189303ASS, 189304ASS, 189330ASS–189338ASS |
|  | Melphalan | 189901ATB, 189902BIJ |
|  | Mercaptopurine | 190601ATB |
|  | Methotrexate | 192101ATB, 192102BIJ–192106BIJ, 192107ATB, 192138BIJ–192143BIJ |
|  | Mitomycin | 196401BIJ–196403BIJ |
|  | Mitoxantrone | 196501BIJ, 196502BIJ, 196530BIJ |
|  | Nilotinib | 562601ACH, 562602ACH |
|  | Nimustine | 202101BIJ |
|  | Nivolumab | 638401BIJ, 638402BIJ |
|  | Obinutuzumab | 628901BIJ |
|  | Olaparib | 643501ACH |
|  | Olaratumab | 658901BIJ |
|  | Osimertinib | 652501ATB, 652502ATB |
|  | Oxaliplatin | 205801BIJ–205805BIJ, 205830BIJ–205834BIJ |
|  | Paclitaxel | 207801BIJ–207805BIJ, 207830BIJ–207835BIJ, 503701BIJ |
|  | Palbociclib | 655201ACH–655203ACH |
|  | Pazopanib | 611801ATB, 611802ATB |
|  | Pembrolizumab | 639001BIJ |
|  | Pemetrexed | 481201BIJ–481205BIJ, 481230BIJ–481234BIJ, 481237BIJ, 481239BIJ |
|  | Pertuzumab | 624601BIJ |
|  | Pirarubicin | 213502BIJ |
|  | Polysaccharide | 215301ACH, 215330AGN, 215302AGN |
|  | Pomalidomide | 628002ACH–628004ACH |
|  | Ponatinib | 669601ATB–669602ATB |
|  | Radotinib | 617701ACH, 617702ACH |
|  | Ramucirumab | 639301BIJ, 639302BIJ |
|  | Regorafenib | 624801ATB |
|  | Rituximab | 422601BIJ–422603BIJ, 422630BIJ–422632BIJ |
|  | Ruxolitinib | 623001ATB–623003ATB |
|  | Sargramostim | 100201BIJ, 100202BIJ |
|  | Siltuximab | 647901BIJ, 647902BIJ |
|  | Sizofiran | 228101BIJ, 228130BIJ |
|  | Sorafenib | 488001ATB |
|  | Streptococcus | 231902BIJ |
|  | Sulfamethoxazole | 311500ATB |
|  | Sunitinib | 487701ACH–487703ACH |
|  | Tacrolimus | 234201ACH, 234203ACH, 234204ACH |
|  | Tamoxifen | 234501ATB, 234502ATB |
|  | Tegafur | 234801ACH |
|  | Temozolomide | 358202ACH–358204ACH |
|  | Temsirolimus | 568230BIJ |
|  | Thalidomide | 485701ACH, 485702ACH |
|  | Thioguanine | 237901ATB |
|  | Thiotepa | 198001BIJ, 198003BIJ |
|  | Topotecan | 241901BIJ, 241902ACH, 241903ACH |
|  | Toremifene | 242101ATB |
|  | Trametinib | 645401ATB, 645402ATB |
|  | Trastuzumab | 242801BIJ–242803BIJ, 242830BIJ, 626001BIJ, 626002BIJ |
|  | Tretinoin | 243001ACS |
|  | Triptorelin | 244901BIJ, 244902BIJ, 244930BIJ, 467501BIJ, 467502BIJ |
|  | Ubenimex | 388501ACH, 388502ACH |
|  | Vandetanib | 624101ATB, 624102ATB |
|  | Vemurafenib | 620501ATB |
|  | Vinblastine | 247801BIJ, 247830BIJ |
|  | Vincristine | 248001BIJ–248003BIJ, 248030BIJ–248032BIJ, 248101BIJ |
|  | Vinorelbine | 248201BIJ, 248202BIJ, 248230BIJ, 248231BIJ |
|  | Viscumalbum | 248502BIJ–248510BIJ, 248601BIJ–248607BIJ |
| Codes for injection of chemotherapy | | |
|  | Anticancer drugs for injection (J0041, KK059, KK151-KK156, KK158, KK159, AP502) | |
| Hematopoietic stem cell transplantation | | |
|  | Allogeneic | X5135, X5133, X5131, X5131100, X5131600, X5133100, X5133600, X5135100, X5135600, X5032, X5033, X5041, X5042, X5011, X5012, X5013, X5014 |
|  | Autologous | X5136, X5134, X5132, X5132100, X5132600, X5134100, X5134600, X5136100, X5136600, X5023, X5024, X5021, X5022 |
| Radiation therapy | | |
|  | HA416, HA441, HA443, HA444, HA445, HA446, HA447, HA448, HD, HX, HY, HZ271 | |
