# Supplementary material for: Comorbidity Differences by Trajectory Groups as a Reference for Identifying Patients at Risk for Late Mortality in Childhood Cancer Survivors: Longitudinal National Cohort Study
Source: JMIR Public Health Surveill. 2023 Mar 24;9:e41203. doi: 10.2196/41203 (PMC10131914; doi:10.2196/41203)
Supplement: Multimedia Appendix 3 [file publichealth_v9i1e41203_app3.docx]

**Multimedia Appendix 3.** Tabulated Bayesian Information Criteria (BIC)’s and 2∆BIC

| **Number of groups** | $\mathbf{BIC}_{\boldsymbol{null}}$ | $\mathbf{BIC}_{\boldsymbol{complex}}$ | **2**$\boldsymbol{\Delta BIC*}$ | **Proportion of group assignment** | | | | |
| --- | --- | --- | --- | --- | --- | --- | --- | --- |
|  |  |  |  | **Group 1** | **Group 2** | **Group 3** | **Group 4** | **Group 5** |
| 1 | -157310 | -157305 | 11.4 | 100 | - | - | - | - |
| 2 | -146000 | -145988 | 23 | 63.9 | 36.1 | - | - | - |
| 3 | -141512 | -141495 | 34.6 | 38.2 | 51.1 | 10.7 | - | - |
| 4 | -139217 | -139194 | 46 | 26.2 | 50.8 | 20.7 | 2.3 | - |
| 5 | -137874 | -137845 | 57.6 | 26.8 | 16.7 | 35.6 | 18.8 | 2.1 |

*∆BIC = $\mathrm{BIC}_{complex}$ - $\mathrm{BIC}_{null}$.

0 < 2$\Delta BIC<2$, 2 < 2$\Delta BIC<6$, 6 < 2$\Delta BIC<10$, and 10 < 2$\Delta BIC$indicate the presence of no significant difference, a positive difference, a strong difference, and a very strong difference between the two models, respectively.
