# Supplementary material for: Comorbidity Differences by Trajectory Groups as a Reference for Identifying Patients at Risk for Late Mortality in Childhood Cancer Survivors: Longitudinal National Cohort Study
Source: JMIR Public Health Surveill. 2023 Mar 24;9:e41203. doi: 10.2196/41203 (PMC10131914; doi:10.2196/41203)

**Multimedia Appendix 4.** Group-based trajectory graphs based on the transformed numbers of diagnosis by follow-up year


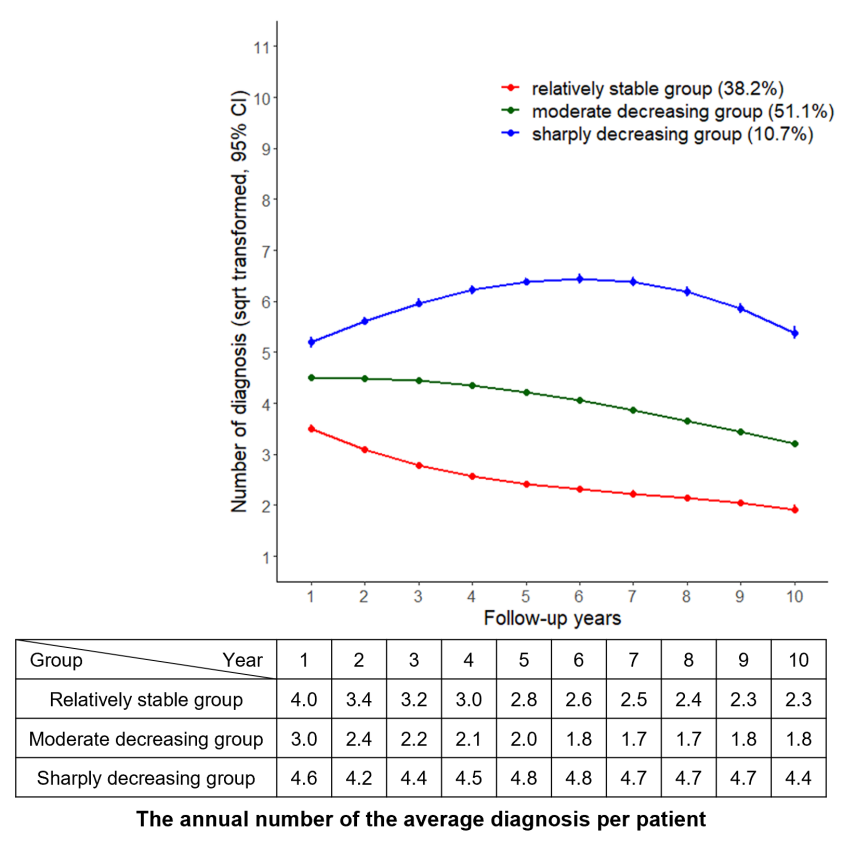

Supplement: Multimedia Appendix 4 [file publichealth_v9i1e41203_app4.docx]
