# Supplementary material for: Comorbidity Differences by Trajectory Groups as a Reference for Identifying Patients at Risk for Late Mortality in Childhood Cancer Survivors: Longitudinal National Cohort Study
Source: JMIR Public Health Surveill. 2023 Mar 24;9:e41203. doi: 10.2196/41203 (PMC10131914; doi:10.2196/41203)
Supplement: Multimedia Appendix 5 [file publichealth_v9i1e41203_app5.docx]

**Multimedia Appendix 5.** Comparison of onset diagnosis and the types of treatment every year after diagnosis

| **Category Year** | | | **2003** | **2004** | **2005** | **2006** | **2007** | **2008** | **Total** | ***P*-value*** |
| --- | --- | --- | --- | --- | --- | --- | --- | --- | --- | --- |
| **Diagnosis** | **Group 1**  (relatively stable) | Central nervous system tumor | 76 (9.4%) | 52 (8.4%) | 60 (12.5%) | 68 (14.9%) | 60 (15%) | 47 (14%) | 363 (11.7%) | .001 |
|  |  | Non-Hodgkin lymphoma | 71 (8.7%) | 73 (11.8%) | 57 (11.9%) | 43 (9.4%) | 62 (15.5%) | 41 (12.2%) | 347 (11.2%) | .012 |
|  |  | Secondary and unspecified malignant neoplasm of lymph nodes | 106 (13.1%) | 71 (11.5%) | 44 (9.2%) | 31 (6.8%) | 22 (5.5%) | 22 (6.5%) | 296 (9.5%) | < .0001 |
|  |  | Total | 812 (100%) | 619 (100%) | 480 (100%) | 457 (100%) | 400 (100%) | 336 (100%) | 3104 (100%) | - |
|  | **Group 2**  (moderately decreasing) | Lymphoid leukemia | 128 (18.2%) | 136 (19.4%) | 150 (20.4%) | 159 (22.7%) | 131 (20.1%) | 134 (20.6%) | 838 (20.2%) | .43 |
|  |  | Central nervous system tumor | 77 (10.9%) | 79 (11.3%) | 73 (9.9%) | 93 (13.3%) | 87 (13.3%) | 92 (14.1%) | 501 (12.1%) | .107 |
|  |  | Non-Hodgkin lymphoma | 68 (9.6%) | 75 (10.7%) | 69 (9.4%) | 54 (7.7%) | 67 (10.3%) | 69 (10.6%) | 402 (9.7%) | .429 |
|  |  | Total | 705 (100%) | 702 (100%) | 736 (100%) | 701 (100%) | 653 (100%) | 651 (100%) | 4148 (100%) | - |
|  | **Group 3**  (sharply  decreasing) | Central nervous system tumor | 8 (9.5%) | 19 (18.6%) | 40 (21.7%) | 33 (20.2%) | 32 (18.7%) | 29 (17.8%) | 161 (18.6%) | .293 |
|  |  | Lymphoid leukemia | 16 (19%) | 18 (17.6%) | 20 (10.9%) | 29 (17.8%) | 36 (21.1%) | 39 (23.9%) | 158 (18.2%) | .047 |
|  |  | Non-Hodgkin lymphoma | 4 (4.8%) | 13 (12.7%) | 15 (8.2%) | 14 (8.6%) | 10 (5.8%) | 20 (12.3%) | 76 (8.8%) | .15 |
|  |  | Total | 84 (100%) | 102 (100%) | 184 (100%) | 163 (100%) | 171 (100%) | 163 (100%) | 867 (100%) | - |
| **Treatment** | **Group 1**  (relatively stable) | CT only | 456 (56.2%) | 328 (53%) | 246 (51.2%) | 200 (43.8%) | 156 (39%) | 93 (27.7%) | 1479 (47.6%) | < .0001 |
|  |  | CT+RT | 300 (36.9%) | 233 (37.6%) | 195 (40.6%) | 187 (40.9%) | 182 (45.5%) | 171 (50.9%) | 1268 (40.9%) | 0 |
|  |  | RT only | 36 (4.4%) | 40 (6.5%) | 34 (7.1%) | 47 (10.3%) | 38 (9.5%) | 36 (10.7%) | 231 (7.4%) | 0 |
|  |  | Total | 812 (100%) | 619 (100%) | 480 (100%) | 457 (100%) | 400 (100%) | 336 (100%) | 3104 (100%) | - |
|  | **Group 2**  (moderately decreasing) | CT+RT | 339 (48.1%) | 398 (56.7%) | 381 (51.8%) | 393 (56.1%) | 378 (57.9%) | 389 (59.8%) | 2278 (54.9%) | < .0001 |
|  |  | CT only | 308 (43.7%) | 229 (32.6%) | 215 (29.2%) | 194 (27.7%) | 162 (24.8%) | 115 (17.7%) | 1223 (29.5%) | < .0001 |
|  |  | CT+RT+allogeneic HSCT | 27 (3.8%) | 29 (4.1%) | 54 (7.3%) | 52 (7.4%) | 54 (8.3%) | 72 (11.1%) | 288 (6.9%) | < .0001 |
|  |  | Total | 705 (100%) | 702 (100%) | 736 (100%) | 701 (100%) | 653 (100%) | 651 (100%) | 4148 (100%) | - |
|  | **Group 3**  (sharply  decreasing) | CT+RT | 45 (53.6%) | 53 (52%) | 91 (49.5%) | 85 (52.1%) | 91 (53.2%) | 85 (52.1%) | 450 (51.9%) | .985 |
|  |  | CT only | 35 (41.7%) | 30 (29.4%) | 48 (26.1%) | 33 (20.2%) | 32 (18.7%) | 19 (11.7%) | 197 (22.7%) | < .0001 |
|  |  | CT+RT+autologous HSCT | 0 (0%) | 6 (5.9%) | 22 (12%) | 15 (9.2%) | 20 (11.7%) | 29 (17.8%) | 92 (10.6%) | 0 |
|  |  | Total | 84 (100%) | 102 (100%) | 184 (100%) | 163 (100%) | 171 (100%) | 163 (100%) | 867 (100%) | - |

*Calculated using the chi-square test.

CT, chemotherapy; RT, radiotherapy; HSCT, hematopoietic stem cell transplantation.
