# Supplementary material for: Comorbidity Differences by Trajectory Groups as a Reference for Identifying Patients at Risk for Late Mortality in Childhood Cancer Survivors: Longitudinal National Cohort Study
Source: JMIR Public Health Surveill. 2023 Mar 24;9:e41203. doi: 10.2196/41203 (PMC10131914; doi:10.2196/41203)
Supplement: Multimedia Appendix 6 [file publichealth_v9i1e41203_app6.docx]

**Multimedia Appendix 6.** International Classification of Diseases (ICD) codes for the top 10 comorbidities

| ICD code | Detailed diagnosis |
| --- | --- |
| A00–A99 | Certain infectious and parasitic diseases |
| B00–B99 | Viral infections and other infectious diseases |
| D00–D89 | Neoplasms and diseases of the blood organs |
| E00–E90 | Endocrine and metabolic diseases |
| F00–F99 | Mental and behavioral disorders |
| H00–H95 | The eye, ear, and adnexa |
| J00–J99 | The respiratory system |
| K00–K93 | The digestive system |
| L00–L99 | The skin and subcutaneous tissue |
| M00–M99 | The musculoskeletal system |
