# Supplementary material for: Comorbidity Differences by Trajectory Groups as a Reference for Identifying Patients at Risk for Late Mortality in Childhood Cancer Survivors: Longitudinal National Cohort Study
Source: JMIR Public Health Surveill. 2023 Mar 24;9:e41203. doi: 10.2196/41203 (PMC10131914; doi:10.2196/41203)
Supplement: Multimedia Appendix 7 [file publichealth_v9i1e41203_app7.docx]

**Multimedia Appendix 7.** Absolute standardized differences between comorbidities of three groups by follow-up (FU) year (for 10 years)

| FU  year | Diagnostic codes | Proportion | | | Chi-square *P*-value  among groups | | | | | Absolute standardized difference | | | | |
| --- | --- | --- | --- | --- | --- | --- | --- | --- | --- | --- | --- | --- | --- | --- |
|  |  | Group 1 | Group 2 | Group 3 | | Group 1  vs. Group 2 | Group 1  vs. Group 3 | Group 2  vs. Group 3 | Group 1  vs. Group 2  vs.  Group 3 | | Group 1  vs. Group 2 | Group 1  vs. Group 3 | Group 2  vs. Group 3 | Max. |
| 1 | Certain infectious and parasitic diseases (A00-A99) | 0.119 | 0.147 | 0.176 | | <.0001 | <.0001 | <.0001 | <.0001 | | 0.082 | 0.161 | 0.079 | 0.161 |
| 2 | Certain infectious and parasitic diseases (A00-A99) | 0.074 | 0.091 | 0.109 | | <.0001 | <.0001 | <.0001 | <.0001 | | 0.062 | 0.119 | 0.057 | 0.119 |
| 3 | Certain infectious and parasitic diseases (A00-A99) | 0.068 | 0.08 | 0.085 | | <.0001 | <.0001 | 0.017 | <.0001 | | 0.045 | 0.064 | 0.019 | 0.064 |
| 4 | Certain infectious and parasitic diseases (A00-A99) | 0.062 | 0.071 | 0.087 | | 0 | <.0001 | <.0001 | <.0001 | | 0.034 | 0.095 | 0.061 | 0.095 |
| 5 | Certain infectious and parasitic diseases (A00-A99) | 0.056 | 0.071 | 0.093 | | <.0001 | <.0001 | <.0001 | <.0001 | | 0.064 | 0.142 | 0.079 | 0.142 |
| 6 | Certain infectious and parasitic diseases (A00-A99) | 0.054 | 0.063 | 0.083 | | <.0001 | <.0001 | <.0001 | <.0001 | | 0.04 | 0.114 | 0.075 | 0.114 |
| 7 | Certain infectious and parasitic diseases (A00-A99) | 0.057 | 0.063 | 0.081 | | 0.006 | <.0001 | <.0001 | <.0001 | | 0.028 | 0.097 | 0.069 | 0.097 |
| 8 | Certain infectious and parasitic diseases (A00-A99) | 0.058 | 0.061 | 0.071 | | 0.254 | <.0001 | <.0001 | <.0001 | | 0.012 | 0.051 | 0.039 | 0.051 |
| 9 | Certain infectious and parasitic diseases (A00-A99) | 0.062 | 0.061 | 0.063 | | 0.662 | 0.737 | 0.317 | 0.587 | | 0.005 | 0.004 | 0.009 | 0.009 |
| 10 | Certain infectious and parasitic diseases (A00-A99) | 0.058 | 0.056 | 0.078 | | 0.382 | <.0001 | <.0001 | <.0001 | | 0.009 | 0.077 | 0.086 | 0.086 |
| 1 | Endocrine and metabolic diseases (E00-E90) | 0.017 | 0.014 | 0.014 | | 0.007 | 0.032 | 0.829 | 0.014 | | 0.023 | 0.025 | 0.003 | 0.025 |
| 2 | Endocrine and metabolic diseases (E00-E90) | 0.018 | 0.013 | 0.013 | | <.0001 | <.0001 | 0.972 | <.0001 | | 0.047 | 0.047 | 0.001 | 0.047 |
| 3 | Endocrine and metabolic diseases (E00-E90) | 0.015 | 0.014 | 0.012 | | 0.22 | 0.031 | 0.169 | 0.089 | | 0.011 | 0.022 | 0.012 | 0.022 |
| 4 | Endocrine and metabolic diseases (E00-E90) | 0.015 | 0.013 | 0.013 | | 0.267 | 0.206 | 0.717 | 0.398 | | 0.01 | 0.013 | 0.003 | 0.013 |
| 5 | Endocrine and metabolic diseases (E00-E90) | 0.012 | 0.016 | 0.018 | | 0.002 | <.0001 | 0.012 | <.0001 | | 0.03 | 0.049 | 0.019 | 0.049 |
| 6 | Endocrine and metabolic diseases (E00-E90) | 0.015 | 0.021 | 0.024 | | <.0001 | <.0001 | 0.007 | <.0001 | | 0.049 | 0.069 | 0.021 | 0.069 |
| 7 | Endocrine and metabolic diseases (E00-E90) | 0.018 | 0.027 | 0.025 | | <.0001 | 0 | 0.056 | <.0001 | | 0.059 | 0.044 | 0.016 | 0.059 |
| 8 | Endocrine and metabolic diseases (E00-E90) | 0.021 | 0.031 | 0.035 | | <.0001 | <.0001 | 0.002 | <.0001 | | 0.062 | 0.087 | 0.025 | 0.087 |
| 9 | Endocrine and metabolic diseases (E00-E90) | 0.025 | 0.041 | 0.04 | | <.0001 | <.0001 | 0.558 | <.0001 | | 0.091 | 0.086 | 0.005 | 0.091 |
| 10 | Endocrine and metabolic diseases (E00-E90) | 0.032 | 0.046 | 0.036 | | <.0001 | 0.047 | <.0001 | <.0001 | | 0.07 | 0.024 | 0.046 | 0.07 |
| 1 | Endocrine and metabolic diseases (E00-E90) | 0.008 | 0.009 | 0.019 | | 0.282 | <.0001 | <.0001 | <.0001 | | 0.01 | 0.1 | 0.091 | 0.1 |
| 2 | Endocrine and metabolic diseases (E00-E90) | 0.014 | 0.015 | 0.026 | | 0.13 | <.0001 | <.0001 | <.0001 | | 0.014 | 0.089 | 0.075 | 0.089 |
| 3 | Mental and behavioral disorders (F00-F99) | 0.014 | 0.016 | 0.035 | | 0.096 | <.0001 | <.0001 | <.0001 | | 0.015 | 0.134 | 0.12 | 0.134 |
| 4 | Mental and behavioral disorders (F00-F99) | 0.013 | 0.015 | 0.035 | | 0.097 | <.0001 | <.0001 | <.0001 | | 0.016 | 0.145 | 0.13 | 0.145 |
| 5 | Mental and behavioral disorders (F00-F99) | 0.017 | 0.019 | 0.038 | | 0.208 | <.0001 | <.0001 | <.0001 | | 0.012 | 0.13 | 0.119 | 0.13 |
| 6 | Mental and behavioral disorders (F00-F99) | 0.017 | 0.022 | 0.05 | | 0 | <.0001 | <.0001 | <.0001 | | 0.036 | 0.185 | 0.151 | 0.185 |
| 7 | Mental and behavioral disorders (F00-F99) | 0.015 | 0.026 | 0.061 | | <.0001 | <.0001 | <.0001 | <.0001 | | 0.08 | 0.242 | 0.17 | 0.242 |
| 8 | Mental and behavioral disorders (F00-F99) | 0.018 | 0.029 | 0.064 | | <.0001 | <.0001 | <.0001 | <.0001 | | 0.074 | 0.234 | 0.166 | 0.234 |
| 9 | Mental and behavioral disorders (F00-F99) | 0.018 | 0.032 | 0.073 | | <.0001 | <.0001 | <.0001 | <.0001 | | 0.087 | 0.263 | 0.184 | 0.263 |
| 10 | Mental and behavioral disorders (F00-F99) | 0.02 | 0.041 | 0.067 | | <.0001 | <.0001 | <.0001 | <.0001 | | 0.121 | 0.231 | 0.115 | 0.231 |
| 1 | Neoplasms or diseases of the blood organs (D00-D89) | 0.088 | 0.073 | 0.087 | | <.0001 | 0.956 | <.0001 | <.0001 | | 0.052 | 0.001 | 0.052 | 0.052 |
| 2 | Neoplasms or diseases of the blood organs (D00-D89) | 0.053 | 0.048 | 0.077 | | 0.005 | <.0001 | <.0001 | <.0001 | | 0.024 | 0.096 | 0.12 | 0.12 |
| 3 | Neoplasms or diseases of the blood organs (D00-D89) | 0.043 | 0.038 | 0.066 | | 0.002 | <.0001 | <.0001 | <.0001 | | 0.027 | 0.102 | 0.128 | 0.128 |
| 4 | Neoplasms or diseases of the blood organs (D00-D89) | 0.032 | 0.033 | 0.07 | | 1 | <.0001 | <.0001 | <.0001 | | 0 | 0.17 | 0.17 | 0.17 |
| 5 | Neoplasms or diseases of the blood organs (D00-D89) | 0.035 | 0.033 | 0.072 | | 0.155 | <.0001 | <.0001 | <.0001 | | 0.013 | 0.165 | 0.178 | 0.178 |
| 6 | Neoplasms or diseases of the blood organs (D00-D89) | 0.037 | 0.034 | 0.081 | | 0.092 | <.0001 | <.0001 | <.0001 | | 0.016 | 0.188 | 0.203 | 0.203 |
| 7 | Neoplasms or diseases of the blood organs (D00-D89) | 0.032 | 0.036 | 0.079 | | 0.013 | <.0001 | <.0001 | <.0001 | | 0.025 | 0.21 | 0.186 | 0.21 |
| 8 | Neoplasms or diseases of the blood organs (D00-D89) | 0.033 | 0.041 | 0.078 | | <.0001 | <.0001 | <.0001 | <.0001 | | 0.042 | 0.196 | 0.156 | 0.196 |
| 9 | Neoplasms or diseases of the blood organs (D00-D89) | 0.028 | 0.043 | 0.096 | | <.0001 | <.0001 | <.0001 | <.0001 | | 0.082 | 0.284 | 0.208 | 0.284 |
| 10 | Neoplasms or diseases of the blood organs (D00-D89) | 0.032 | 0.042 | 0.099 | | <.0001 | <.0001 | <.0001 | <.0001 | | 0.051 | 0.275 | 0.227 | 0.275 |
| 1 | Neoplasms (D00-D49) | 0.056 | 0.037 | 0.037 | | <.0001 | <.0001 | 1 | <.0001 | | 0.092 | 0.092 | 0 | 0.092 |
| 2 | Neoplasms (D00-D49) | 0.032 | 0.022 | 0.047 | | <.0001 | <.0001 | <.0001 | <.0001 | | 0.061 | 0.048 | 0.108 | 0.108 |
| 3 | Neoplasms (D00-D49) | 0.026 | 0.018 | 0.035 | | <.0001 | <.0001 | <.0001 | <.0001 | | 0.055 | 0.051 | 0.105 | 0.105 |
| 4 | Neoplasms (D00-D49) | 0.019 | 0.016 | 0.043 | | 0.004 | <.0001 | <.0001 | <.0001 | | 0.026 | 0.141 | 0.165 | 0.165 |
| 5 | Neoplasms (D00-D49) | 0.022 | 0.017 | 0.044 | | 0 | <.0001 | <.0001 | <.0001 | | 0.034 | 0.127 | 0.158 | 0.158 |
| 6 | Neoplasms (D00-D49) | 0.027 | 0.018 | 0.042 | | <.0001 | <.0001 | <.0001 | <.0001 | | 0.061 | 0.084 | 0.143 | 0.143 |
| 7 | Neoplasms (D00-D49) | 0.022 | 0.023 | 0.042 | | 0.392 | <.0001 | <.0001 | <.0001 | | 0.009 | 0.116 | 0.107 | 0.142 |
| 8 | Neoplasms (D00-D49) | 0.02 | 0.025 | 0.052 | | 0.001 | <.0001 | <.0001 | <.0001 | | 0.034 | 0.174 | 0.143 | 0.116 |
| 9 |  |  |  |  | |  |  |  |  | |  |  |  | 0.174 |
| 10 | Neoplasms (D00-D49) | 0.023 | 0.023 | 0.047 | | 0.742 | <.0001 | <.0001 | <.0001 | | 0.004 | 0.13 | 0.133 | 0.133 |
| 1 | The diseases of the blood organs (D50-D89) | 0.031 | 0.036 | 0.05 | | 0.001 | <.0001 | <.0001 | <.0001 | | 0.028 | 0.096 | 0.069 | 0.096 |
| 2 | The diseases of the blood organs (D50-D89) | 0.022 | 0.026 | 0.037 | | 0.001 | <.0001 | <.0001 | <.0001 | | 0.03 | 0.088 | 0.059 | 0.088 |
| 3 | The diseases of the blood organs (D50-D89) | 0.017 | 0.02 | 0.031 | | 0.021 | <.0001 | <.0001 | <.0001 | | 0.021 | 0.094 | 0.073 | 0.094 |
| 4 | The diseases of the blood organs (D50-D89) | 0.013 | 0.017 | 0.026 | | 0.003 | <.0001 | <.0001 | <.0001 | | 0.028 | 0.092 | 0.065 | 0.092 |
| 5 | The diseases of the blood organs (D50-D89) | 0.013 | 0.016 | 0.028 | | 0.05 | <.0001 | <.0001 | <.0001 | | 0.019 | 0.103 | 0.084 | 0.103 |
| 6 | The diseases of the blood organs (D50-D89) | 0.01 | 0.016 | 0.039 | | <.0001 | <.0001 | <.0001 | <.0001 | | 0.053 | 0.187 | 0.139 | 0.187 |
| 7 | The diseases of the blood organs (D50-D89) | 0.009 | 0.018 | 0.037 | | <.0001 | <.0001 | <.0001 | <.0001 | | 0.081 | 0.19 | 0.116 | 0.19 |
| 8 | The diseases of the blood organs (D50-D89) | 0.011 | 0.018 | 0.036 | | <.0001 | <.0001 | <.0001 | <.0001 | | 0.054 | 0.161 | 0.111 | 0.161 |
| 9 | The diseases of the blood organs (D50-D89) | 0.008 | 0.018 | 0.044 | | <.0001 | <.0001 | <.0001 | <.0001 | | 0.089 | 0.224 | 0.146 | 0.224 |
| 10 | The diseases of the blood organs (D50-D89) | 0.008 | 0.019 | 0.052 | | <.0001 | <.0001 | <.0001 | <.0001 | | 0.088 | 0.256 | 0.181 | 0.256 |
| 1 | The diseases of the digestive system (K00-K93) | 0.045 | 0.033 | 0.022 | | <.0001 | <.0001 | <.0001 | <.0001 | | 0.065 | 0.127 | 0.063 | 0.127 |
| 2 | The diseases of the digestive system (K00-K93) | 0.055 | 0.036 | 0.031 | | <.0001 | <.0001 | 0.003 | <.0001 | | 0.089 | 0.116 | 0.028 | 0.116 |
| 3 | The diseases of the digestive system (K00-K93) | 0.069 | 0.042 | 0.023 | | <.0001 | <.0001 | <.0001 | <.0001 | | 0.116 | 0.221 | 0.109 | 0.221 |
| 4 | The diseases of the digestive system (K00-K93) | 0.078 | 0.045 | 0.026 | | <.0001 | <.0001 | <.0001 | <.0001 | | 0.139 | 0.235 | 0.101 | 0.235 |
| 5 | The diseases of the digestive system (K00-K93) | 0.094 | 0.055 | 0.03 | | <.0001 | <.0001 | <.0001 | <.0001 | | 0.148 | 0.267 | 0.124 | 0.267 |
| 6 | The diseases of the digestive system (K00-K93) | 0.113 | 0.07 | 0.036 | | <.0001 | <.0001 | <.0001 | <.0001 | | 0.151 | 0.294 | 0.149 | 0.294 |
| 7 | The diseases of the digestive system (K00-K93) | 0.125 | 0.08 | 0.043 | | <.0001 | <.0001 | <.0001 | <.0001 | | 0.148 | 0.298 | 0.154 | 0.298 |
| 8 | The diseases of the digestive system (K00-K93) | 0.155 | 0.098 | 0.053 | | <.0001 | <.0001 | <.0001 | <.0001 | | 0.172 | 0.339 | 0.171 | 0.339 |
| 9 | The diseases of the digestive system (K00-K93) | 0.156 | 0.109 | 0.062 | | <.0001 | <.0001 | <.0001 | <.0001 | | 0.139 | 0.307 | 0.171 | 0.307 |
| 10 | The diseases of the digestive system (K00-K93) | 0.162 | 0.114 | 0.06 | | <.0001 | <.0001 | <.0001 | <.0001 | | 0.138 | 0.33 | 0.195 | 0.33 |
| 1 | The diseases of the eye, ear, and adnexa (H00-H95) | 0.122 | 0.117 | 0.124 | | 0.082 | 0.534 | 0.032 | 0.048 | | 0.015 | 0.007 | 0.022 | 0.022 |
| 2 | The diseases of the eye, ear, and adnexa (H00-H95) | 0.147 | 0.153 | 0.162 | | 0.068 | 0 | 0.006 | 0.001 | | 0.016 | 0.042 | 0.025 | 0.042 |
| 3 | The diseases of the eye, ear, and adnexa (H00-H95) | 0.147 | 0.164 | 0.187 | | <.0001 | <.0001 | <.0001 | <.0001 | | 0.047 | 0.107 | 0.06 | 0.107 |
| 4 | The diseases of the eye, ear, and adnexa (H00-H95) | 0.156 | 0.156 | 0.174 | | 0.927 | <.0001 | <.0001 | <.0001 | | 0.001 | 0.047 | 0.046 | 0.047 |
| 5 | The diseases of the eye, ear, and adnexa (H00-H95) | 0.151 | 0.145 | 0.157 | | 0.097 | 0.085 | <.0001 | <.0001 | | 0.016 | 0.018 | 0.033 | 0.033 |
| 6 | The diseases of the eye, ear, and adnexa (H00-H95) | 0.155 | 0.144 | 0.145 | | 0.002 | 0.006 | 0.977 | 0.006 | | 0.029 | 0.029 | 0 | 0.029 |
| 7 | The diseases of the eye, ear, and adnexa (H00-H95) | 0.141 | 0.143 | 0.132 | | 0.773 | 0.01 | 0 | 0 | | 0.003 | 0.028 | 0.031 | 0.031 |
| 8 | The diseases of the eye, ear, and adnexa (H00-H95) | 0.148 | 0.138 | 0.138 | | 0.004 | 0.011 | 0.924 | 0.011 | | 0.029 | 0.028 | 0.001 | 0.029 |
| 9 | The diseases of the eye, ear, and adnexa (H00-H95) | 0.143 | 0.128 | 0.133 | | <.0001 | 0.01 | 0.116 | 0 | | 0.043 | 0.029 | 0.014 | 0.043 |
| 10 | The diseases of the eye, ear, and adnexa (H00-H95) | 0.146 | 0.13 | 0.125 | | <.0001 | <.0001 | 0.101 | <.0001 | | 0.046 | 0.061 | 0.015 | 0.061 |
| 1 | The diseases of the musculoskeletal system and connective tissue (M00-M99) | 0.025 | 0.017 | 0.014 | | <.0001 | <.0001 | 0.025 | <.0001 | | 0.057 | 0.081 | 0.024 | 0.081 |
| 2 | The diseases of the musculoskeletal system and connective tissue (M00-M99) | 0.026 | 0.021 | 0.014 | | 0 | <.0001 | <.0001 | <.0001 | | 0.033 | 0.089 | 0.056 | 0.089 |
| 3 | The diseases of the musculoskeletal system and connective tissue (M00-M99) | 0.033 | 0.021 | 0.016 | | <.0001 | <.0001 | <.0001 | <.0001 | | 0.073 | 0.108 | 0.036 | 0.108 |
| 4 | The diseases of the musculoskeletal system and connective tissue (M00-M99) | 0.034 | 0.025 | 0.022 | | <.0001 | <.0001 | 0.048 | <.0001 | | 0.054 | 0.07 | 0.015 | 0.07 |
| 5 | The diseases of the musculoskeletal system and connective tissue (M00-M99) | 0.036 | 0.03 | 0.034 | | 0 | 0.489 | 0 | <.0001 | | 0.034 | 0.007 | 0.027 | 0.034 |
| 6 | The diseases of the musculoskeletal system and connective tissue (M00-M99) | 0.041 | 0.034 | 0.042 | | 0 | 0.514 | <.0001 | <.0001 | | 0.034 | 0.007 | 0.042 | 0.042 |
| 7 | The diseases of the musculoskeletal system and connective tissue (M00-M99) | 0.043 | 0.034 | 0.046 | | <.0001 | 0.125 | <.0001 | <.0001 | | 0.044 | 0.017 | 0.061 | 0.061 |
| 8 | The diseases of the musculoskeletal system and connective tissue (M00-M99) | 0.054 | 0.042 | 0.06 | | <.0001 | 0.026 | <.0001 | <.0001 | | 0.055 | 0.025 | 0.079 | 0.079 |
| 9 | The diseases of the musculoskeletal system and connective tissue (M00-M99) | 0.056 | 0.043 | 0.057 | | <.0001 | 0.659 | <.0001 | <.0001 | | 0.06 | 0.005 | 0.066 | 0.066 |
| 10 | The diseases of the musculoskeletal system and connective tissue (M00-M99) | 0.054 | 0.05 | 0.056 | | 0.133 | 0.399 | 0.004 | 0.013 | | 0.016 | 0.01 | 0.026 | 0.026 |
| 1 | The diseases of the respiratory system (J00-J99) | 0.351 | 0.316 | 0.301 | | <.0001 | <.0001 | 0.003 | <.0001 | | 0.075 | 0.106 | 0.031 | 0.106 |
| 2 | The diseases of the respiratory system (J00-J99) | 0.453 | 0.453 | 0.423 | | 0.899 | <.0001 | <.0001 | <.0001 | | 0.001 | 0.06 | 0.059 | 0.06 |
| 3 | The diseases of the respiratory system (J00-J99) | 0.471 | 0.499 | 0.477 | | <.0001 | 0.203 | <.0001 | <.0001 | | 0.058 | 0.013 | 0.044 | 0.058 |
| 4 | The diseases of the respiratory system (J00-J99) | 0.478 | 0.532 | 0.49 | | <.0001 | 0.02 | <.0001 | <.0001 | | 0.109 | 0.024 | 0.085 | 0.109 |
| 5 | The diseases of the respiratory system (J00-J99) | 0.482 | 0.537 | 0.481 | | <.0001 | 0.847 | <.0001 | <.0001 | | 0.11 | 0.002 | 0.112 | 0.112 |
| 6 | The diseases of the respiratory system (J00-J99) | 0.457 | 0.516 | 0.46 | | <.0001 | 0.607 | <.0001 | <.0001 | | 0.118 | 0.006 | 0.112 | 0.118 |
| 7 | The diseases of the respiratory system (J00-J99) | 0.45 | 0.489 | 0.452 | | <.0001 | 0.69 | <.0001 | <.0001 | | 0.079 | 0.004 | 0.074 | 0.079 |
| 8 | The diseases of the respiratory system (J00-J99) | 0.394 | 0.459 | 0.411 | | <.0001 | 0.003 | <.0001 | <.0001 | | 0.131 | 0.033 | 0.098 | 0.131 |
| 9 | The diseases of the respiratory system (J00-J99) | 0.379 | 0.435 | 0.378 | | <.0001 | 0.902 | <.0001 | <.0001 | | 0.114 | 0.002 | 0.116 | 0.116 |
| 10 | The diseases of the respiratory system (J00-J99) | 0.365 | 0.414 | 0.384 | | <.0001 | 0.001 | <.0001 | <.0001 | | 0.101 | 0.039 | 0.063 | 0.101 |
| 1 | The diseases of the skin and subcutaneous tissue (L00-L99) | 0.051 | 0.041 | 0.033 | | <.0001 | <.0001 | <.0001 | <.0001 | | 0.047 | 0.09 | 0.043 | 0.09 |
| 2 | The diseases of the skin and subcutaneous tissue (L00-L99) | 0.057 | 0.046 | 0.037 | | <.0001 | <.0001 | <.0001 | <.0001 | | 0.049 | 0.095 | 0.047 | 0.095 |
| 3 | The diseases of the skin and subcutaneous tissue (L00-L99) | 0.061 | 0.042 | 0.028 | | <.0001 | <.0001 | <.0001 | <.0001 | | 0.086 | 0.163 | 0.079 | 0.163 |
| 4 | The diseases of the skin and subcutaneous tissue (L00-L99) | 0.069 | 0.046 | 0.028 | | <.0001 | <.0001 | <.0001 | <.0001 | | 0.097 | 0.193 | 0.098 | 0.193 |
| 5 | The diseases of the skin and subcutaneous tissue (L00-L99) | 0.063 | 0.044 | 0.03 | | <.0001 | <.0001 | <.0001 | <.0001 | | 0.086 | 0.157 | 0.072 | 0.157 |
| 6 | The diseases of the skin and subcutaneous tissue (L00-L99) | 0.067 | 0.049 | 0.031 | | <.0001 | <.0001 | <.0001 | <.0001 | | 0.079 | 0.168 | 0.09 | 0.168 |
| 7 | The diseases of the skin and subcutaneous tissue (L00-L99) | 0.071 | 0.053 | 0.033 | | <.0001 | <.0001 | <.0001 | <.0001 | | 0.075 | 0.173 | 0.1 | 0.173 |
| 8 | The diseases of the skin and subcutaneous tissue (L00-L99) | 0.069 | 0.056 | 0.037 | | <.0001 | <.0001 | <.0001 | <.0001 | | 0.057 | 0.143 | 0.087 | 0.143 |
| 9 | The diseases of the skin and subcutaneous tissue (L00-L99) | 0.072 | 0.058 | 0.042 | | <.0001 | <.0001 | <.0001 | <.0001 | | 0.058 | 0.129 | 0.072 | 0.129 |
| 10 | The diseases of the skin and subcutaneous tissue (L00-L99) | 0.073 | 0.059 | 0.04 | | <.0001 | <.0001 | <.0001 | <.0001 | | 0.055 | 0.145 | 0.091 | 0.145 |
| 1 | Viral infections and other infectious diseases (B00-B99) | 0.174 | 0.233 | 0.209 | | <.0001 | <.0001 | <.0001 | <.0001 | | 0.148 | 0.088 | 0.06 | 0.148 |
| 2 | Viral infections and other infectious diseases (B00-B99) | 0.103 | 0.124 | 0.109 | | <.0001 | 0.068 | <.0001 | <.0001 | | 0.067 | 0.02 | 0.047 | 0.067 |
| 3 | Viral infections and other infectious diseases (B00-B99) | 0.08 | 0.084 | 0.071 | | 0.08 | 0.001 | <.0001 | <.0001 | | 0.016 | 0.034 | 0.049 | 0.049 |
| 4 | Viral infections and other infectious diseases (B00-B99) | 0.064 | 0.065 | 0.056 | | 0.652 | 0.001 | <.0001 | <.0001 | | 0.004 | 0.033 | 0.037 | 0.037 |
| 5 | Viral infections and other infectious diseases (B00-B99) | 0.054 | 0.051 | 0.046 | | 0.076 | <.0001 | 0.002 | 0 | | 0.017 | 0.04 | 0.024 | 0.04 |
| 6 | Viral infections and other infectious diseases (B00-B99) | 0.045 | 0.047 | 0.048 | | 0.256 | 0.15 | 0.587 | 0.339 | | 0.011 | 0.015 | 0.004 | 0.015 |
| 7 | Viral infections and other infectious diseases (B00-B99) | 0.048 | 0.048 | 0.047 | | 0.929 | 0.83 | 0.866 | 0.969 | | 0.001 | 0.003 | 0.002 | 0.003 |
| 8 | Viral infections and other infectious diseases (B00-B99) | 0.05 | 0.046 | 0.054 | | 0.077 | 0.078 | <.0001 | <.0001 | | 0.018 | 0.02 | 0.038 | 0.038 |
| 9 | Viral infections and other infectious diseases (B00-B99) | 0.061 | 0.05 | 0.056 | | <.0001 | 0.109 | 0 | <.0001 | | 0.048 | 0.018 | 0.03 | 0.048 |
| 10 | Viral infections and other infectious diseases (B00-B99) | 0.058 | 0.047 | 0.056 | | <.0001 | 0.337 | <.0001 | <.0001 | | 0.048 | 0.012 | 0.037 | 0.048 |
