# Supplementary material for: Comorbidity Differences by Trajectory Groups as a Reference for Identifying Patients at Risk for Late Mortality in Childhood Cancer Survivors: Longitudinal National Cohort Study
Source: JMIR Public Health Surveill. 2023 Mar 24;9:e41203. doi: 10.2196/41203 (PMC10131914; doi:10.2196/41203)
Supplement: Multimedia Appendix 8 [file publichealth_v9i1e41203_app8.docx]

**Multimedia Appendix 8.** Diagnosis at the time of death

| **Cause of death**  **(ICD code)** | **Group 1**  **(relatively stable)** | **Group 2**  **(moderately decreasing)** | **Group 3**  **(sharply**  **decreasing)** | **Total** |
| --- | --- | --- | --- | --- |
| ***Top 10 leading causes, n (%)*** | 25 (100%) | 65 (100%) | 43 (100%) | 133 (100%) |
| Brain and CNS (C71) | 5 (20%) | 13 (20%) | 8 (18.6%) | 26 (19.5%) |
| Lymphoid leukemia (C91) | 0 (0%) | 7 (10.8%) | 9 (20.9%) | 16 (12%) |
| Injury, poisoning, and certain other consequences of external causes (S00-T98) | 5 (20%) | 6 (9.2%) | 0 (0%) | 11 (8.3%) |
| Unknown | 2 (8%) | 6 (9.2%) | 2 (4.7%) | 10 (7.5%) |
| Myeloid leukemia (C92) | 2 (8%) | 5 (7.7%) | 2 (4.7%) | 9 (6.8%) |
| Bone (C40) | 2 (8%) | 3 (4.6%) | 2 (4.7%) | 7 (5.3%) |
| Connective and soft tissue (C49) | 1 (4%) | 0 (0%) | 4 (9.3%) | 5 (3.8%) |
| Myelodysplastic syndromes (D46) | 0 (0%) | 2 (3.1%) | 1 (2.3%) | 3 (2.3%) |
| Bone (C41) | 0 (0%) | 0 (0%) | 3 (7%) | 3 (2.3%) |
| Ill-defined and unknown causes of mortality (R99) | 1 (4%) | 2 (3.1%) | 0 (0%) | 3 (2.3%) |

ICD, International Classification of Diseases; CNS, central nervous system
